# A Simple CO_2_ Generating System Incorporated with CDC Light Trap for Sampling Mosquito Vectors

**DOI:** 10.3390/insects13070637

**Published:** 2022-07-15

**Authors:** Sutasinee Madang, Jassada Saingamsook, Atiporn Saeung, Pradya Somboon

**Affiliations:** 1 Graduate Master’s Degree Program in Parasitology, Faculty of Medicine, Chiang Mai University, Chiang Mai 50200, Thailand; sutasinee_madang@cmu.ac.th; 2 Center of Insect Vector Study, Department of Parasitology, Faculty of Medicine, Chiang Mai University, Chiang Mai 50200, Thailand; atiporn.s@cmu.ac.th (A.S.); pradya.somboon@cmu.ac.th (P.S.)

**Keywords:** mosquito collection, CDC trap, CO_2_ trap, HCl, limestone, CaCO_3_

## Abstract

**Simple Summary:**

This study successfully developed a CO_2_ generating system that can be incorporated with a CDC light trap for the overnight collection of mosquitoes. We produced CO_2_ continuously by dripping an aqueous solution of 12% *w*/*w* hydrochloric acid (HCl) (30 drops or 1.6 mL/min) controlled by an intravenous drip infusion set onto limestone powder (800 g) that produced an average of 55 mL CO_2_/min (equivalent to the CO_2_ exhalation from two chickens). The efficiency of this trap set for capturing mosquitoes was evaluated in the field compared with the light trap alone and the light trap baited with 1 kg dry ice. The results revealed that the trap with the acid and limestone significantly increased the number and species composition of mosquitoes collected compared with the light trap alone. It could collect all important vector species of *Aedes*, *Armigeres*, *Coquilletidia*, *Culex* and *Mansonia* as collected by the trap with dry ice, although the numbers were fewer. Our CO_2_ producing system is reliable, simple and inexpensive, and could be an alternative method when dry ice is unavailable.

**Abstract:**

Traps for capturing mosquitoes and other blood-feeding arthropods are often baited with carbon dioxide (CO_2_) as an attractant. Dry ice is popularly used as a CO_2_ source due to its high efficiency and ease of use. However, dry ice can be difficult to obtain in many rural and remote areas. The objective of this study was to develop a simple and inexpensive method that could continuously generate CO_2_ overnight (about 10 h) while being used with CDC light traps for sampling adult mosquitoes. In principle, CO_2_ was produced from the reaction between hydrochloric acid (HCl) (12% *w*/*w*) and limestone powder (mainly composed of calcium carbonate, CaCO_3_). In laboratory experiments, an average of 256 mL of CO_2_ was produced from 1 g of limestone. For continuous production of CO_2_, an intravenous drip infusion set, as commonly used in hospitals, was modified for dripping the acid solution (1 L in a normal saline bag) onto limestone powder (800 g in a 1.5 L bottle) at a flow rate of 30 drops/min (about 1.6 mL/min). With this procedure, an average of 55 mL of CO_2_ per min was obtained (approximately equivalent to the CO_2_ exhaled by two chickens). The performance of this CO_2_ generating system incorporated with CDC light traps for sampling mosquitoes was evaluated in three rural villages of Sanpatong District, Chiang Mai Province, Thailand. Three trap sets were used, i.e., Set I, light trap alone; Set II, light trap with dry ice (1 kg); and Set III, light trap with limestone and acid. In each village, mosquitoes were collected at three fixed sites, each with one of the three trap sets. They were rotated daily for three rounds (9 nights per village and 27 nights in total). A total of 1620 mosquitoes (97.7% being females) consisting of *Aedes*, *Anopheles*, *Armigeres*, *Coquilletidia*, *Culex* and *Mansonia* were captured across three different sampling sets from all villages. The predominant species collected were *Culex vishnui* (*n* = 760, 46.91%), *Cx. bitaeniorhynchus* (*n* = 504, 31.11%) and *Cx. tritaeniorhynchus* (*n* = 157, 9.69%). Light traps alone (Set I) collected very low numbers of mosquitoes (*n* = 12) and species (6 spp.), whereas light traps with dry ice (Set II) collected the highest numbers of mosquitoes (*n* = 1341) and species (14 spp.). Although the light trap with limestone and acid (Set III) collected fewer mosquitoes (*n* = 267) and species (9 spp.) than the trap set with dry ice (Set II), it collected all common vector species in the study areas as collected by Set II. The presence of an acid solution had no bias in the collection of mosquitoes with different physiological ages as determined by the parous rate. The present study demonstrated that this CO_2_ generating system is reliable, simple and inexpensive, and could be an alternative to dry ice. The system can be modified to increase the amount of CO_2_ generated for higher efficacy of mosquito collection. This CO_2_ production method can be applied to collect other blood-sucking arthropods as well.

## 1. Introduction

Carbon dioxide produced by breathing is known to be an important factor in attracting mosquitoes and other hematophagous arthropods to their hosts. Carbon dioxide can be added to various traps, such as the CDC light trap, to increase collection effectiveness. Several studies have shown that carbon dioxide traps are powerful tools for sampling adult mosquitoes, which is important for vector surveillance and control programs [1]. Generally, carbon dioxide can be obtained in the form of gas in cylinders or through the sublimation of dry ice. However, the uniform release of gas from the cylinder necessitates a sensitive regulatory valve system and meters to control and measure flow rates. These, together with the cylinders, are more costly and bulky than dry ice. In certain areas, however, dry ice may be difficult to obtain. Its losses due to sublimation during transportation and maintenance in the field can increase costs.

Alternative carbon dioxide sources for mosquito attractants have been produced, including burning petroleum gases (e.g., propane or LPG) [2]. This system has been applied to some commercial traps [3]. However, a device with a liquid petroleum tank is rather expensive and heavy. Saito et al. [4] developed a convenient source of carbon dioxide by fermentation of sugar with yeast generating an average of 32.4 mL/min, nearly equal to the output rate of a chicken. Steiger et al. [5] showed that a maximum average of about 54 mL/min carbon dioxide could be produced by 30 g yeast + 250 g sugar in 1 L of water at 30 °C. Although the flow rate of yeast-generated carbon dioxide is about 10 times lower than that of a kilogram of dry ice, resulting in fewer mosquitoes collected, there was little effect on mosquito species composition [4,5]. Fermentation, however, requires some time before the desired amount of carbon dioxide is generated. In addition, the amount of carbon dioxide generated by yeast depends on temperature and, thus, is affected by cold weather.

Carbon dioxide can be produced as an attractant through the reactions of weak acid and carbonate, e.g., acetic acid (CH_3_COOH) + sodium bicarbonate (NaHCO_3_) [6], or lactic acid (C_3_H_6_O_3_) + calcium carbonate (CaCO_3_) [7,8]. This method has been mostly used for collecting ticks. However, this method may not be applicable for mosquito collection as these weak acids do not produce a sufficiently continuous amount of carbon dioxide throughout the night. Moreover, the cost of these reagents (e.g., sodium bicarbonate and weak acids, e.g., acetic acid, citric acid, malic acid and lactic acid) and the necessity of a large quantity of water for preparing the acid solution are particularly disadvantageous when collections are made in remote areas [9]. Attempts to reduce the cost and increase the efficacy of trap collection have been made by using inexpensive carbonate and an electronic device to control the flow of CO_2_ output. Burkett-Cadena et al. [10] developed a CO_2_ generating device that used calcium carbonate derived from crushed coquina and shell gravel combined with 5% acetic acid (C_2_H_4_O_2_) or 10% citric acid (C_6_H_8_O_7_). This device was used in conjunction with CDC light traps for mosquito collection. Both the carbonate and acid solution were mixed at one time in a container, which produced roughly 200–300 mL/min of CO_2_ at the moment of peak production (15–20 min). However, the volume of CO_2_ produced diminished substantially to around 50 mL/min by two hours and reduced to 10–20 mL/min by four hours. In addition, the authors also used a programmable hose-end sprinkler timer to control the timing and duration of the flow of citric acid solution dripping onto the sodium bicarbonate. The flow of citric acid solution was 36.5 mL/min at initiation, but this flow rate slowed incrementally over time, by the variable rate of approximately 0.3 mL/min for each minute, over the entire flow period. CO_2_ production from the automated system peaked within the first 10 min of the initiation of the reaction, at approximately 550 mL/min. The decline of CO_2_ output was relatively steady, with about 150 mL/min after four hours, but no observation was done after that. The number of female mosquitoes collected in traps paired with CO_2_ from crushed shells or sodium carbonate and citric acid was 70% lower than in traps using dry ice as an attractant. Still, the composition of common mosquito species was similar [10].

It can be seen from the previous studies that CO_2_ generated by mixing carbonates and weak acid solution at one time was produced quickly and reached the peak within a short time, then followed by a significant decrease in gas. This method cannot maintain the desired level of CO_2_ throughout the night and may not be able to collect species that normally come late at night or early morning. Therefore, this study aimed to develop a CO_2_ generating system using a strong acid (either hydrochloric acid (HCl) or sulfuric acid (H_2_SO_4_)) and limestone (known to be composed mainly of calcium carbonate (CaCO_3_)) [11], and evaluated its efficacy compared with dry ice for the overnight sampling of mosquitoes with CDC light traps. We also evaluated the effect of hydrochloric acid on collecting mosquitoes with different age structures since the presence of acid vapor was reported to have a repellent effect on female flies and gravid mosquitoes [12,13]. These acids and limestone are cheap and widely available in Thailand. Additionally, limestone can be purchased in various forms and quantities.

## 2. Materials and Methods

### 2.1. CO_2_ Production from Limestone

**Figure 1:**
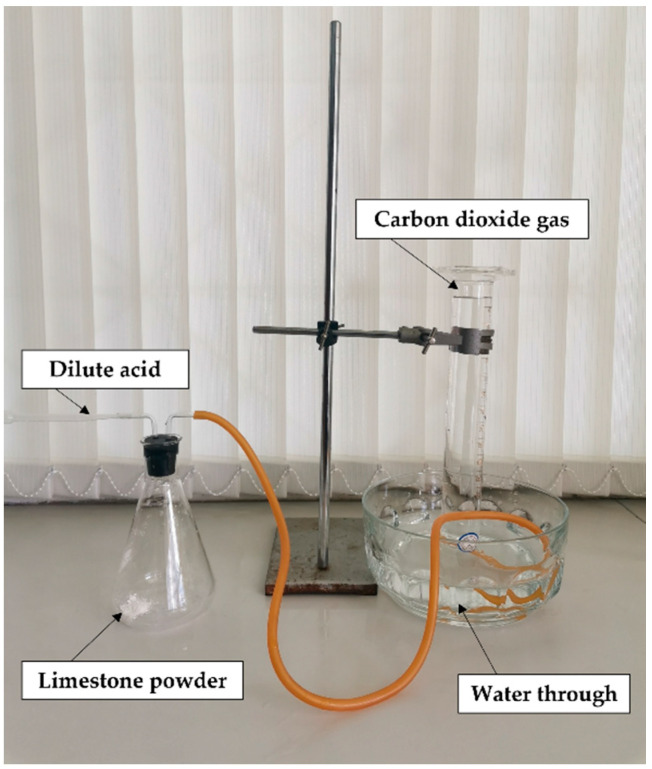
Determination of CO_2_ production from limestone and acid reaction.

Limestone was purchased (2 USD per 50 kg) as a powder from a company in Lampang Province, northern Thailand. This form is commonly used to improve soil quality for agricultural purposes. It is generally known that limestone is composed principally of calcium carbonate, but its quantity may vary depending on the chemical composition of limestone. When reacted with an acid, it is reasonable to believe that most of the gas output is CO_2_, although there might be some other gases produced, but, if any, this is considered very minor and has little effect on the present study. We hereby call the output gas as CO_2_. Initially, it was necessary to know the quantity of limestone-produced CO_2_ when reacted with an acid solution by measuring gas output compared with the same amount of calcium carbonate (>99% purity, industrial grade, Union Science, Chiang Mai, Thailand), which was used as control material. This experiment aimed to measure the amount of gas, assuming mostly CO_2_, produced from one gram of limestone under excess acid solution in order to estimate the amount of limestone to be used for overnight gas production. Two strong acids, H_2_SO_4_ and HCl, were evaluated, and only one acid with superior performance was selected for use in field trials. An excess volume of 15% *w*/*w* of H_2_SO_4_ (prepared from conc. H_2_SO_4_ 98% *w*/*w* (RCI Labscan, Bangkok, Thailand)) and 10% and 12% *w*/*w* of HCl (prepared from conc. HCl 37% *w*/*w* (RCI Labscan, Bangkok, Thailand)) was added to one gram of limestone powder or calcium carbonate powder in a bottle connected with two rubber tubes, one for the input of acid solution and the other for output of gas (Figure 1). The reason we used lower concentrations is that HCl has higher acidity (based on pKa value) than H_2_SO_4_ to donate a proton. We did not use 15% HCL solution because it is more hazardous when used in the field. CO_2_ output per gram of limestone was determined by measuring the volume of water displaced in a submerged measuring cylinder until the gas was no longer produced (about 15 min). The absolute volume of gas was derived by deducting the total gas volume with the volume of acid solution added. The amount of limestone and acid solution to be used for the overnight collection was calculated from the output of CO_2_ gas above.

### 2.2. CO_2_ Generating System for Overnight Collection

**Figure 2:**
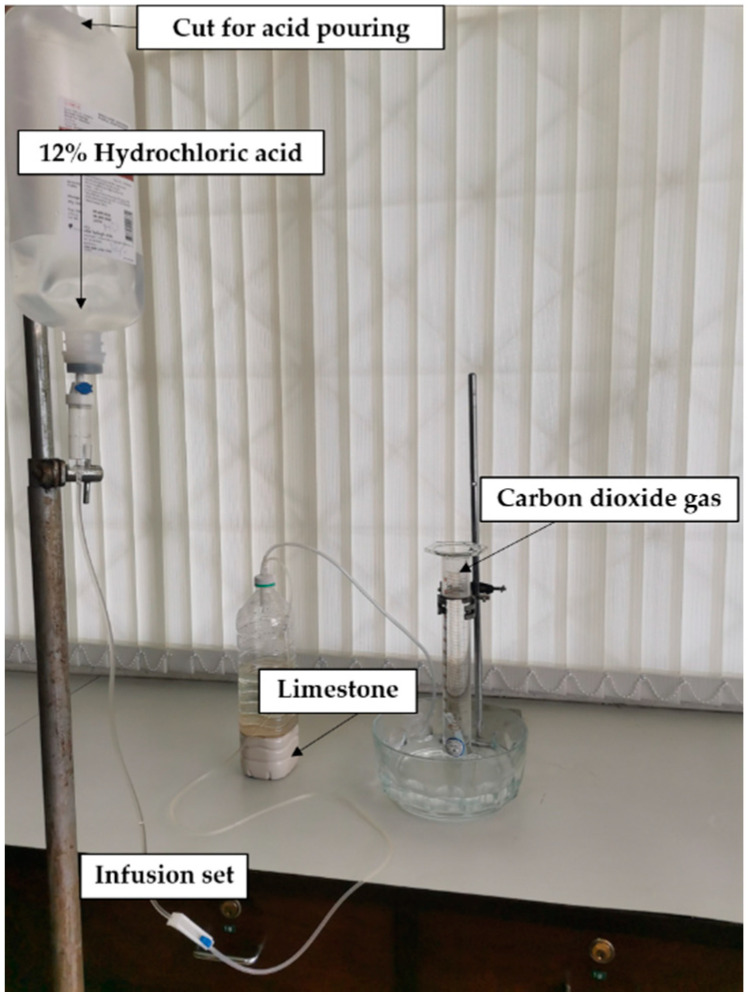
Determination of CO_2_ flow from limestone reacted with continuous dripping of acid solution.

For the overnight collection of mosquitoes, a continuous flow of CO_2_ at a desired level throughout the collection time (about 10–12 h) is essential. The target CO_2_ output was about 50–70 mL/min, which is about twice as much as that produced by the reaction of yeast and sugar or that exhaled from a chick [4]. The production of CO_2_ per min depends on the type of acid, concentration and flow rate of acid solution. The flow of acid solution was controlled by using an intravenous tube with a roller clamp on the tube, which is connected to a 1 L plastic bottle (Thai-Otsuka Pharmaceutical Co., Ltd., Bangkok, Thailand); its bottom was cut (about 2 inches long) for the pouring of acid solution (Figure 2). The flow rate of acid was 30 drops (about 1.6 mL) per minute (or 96 mL per hour). HCl was used due to its greater performance than H_2_SO_4_ in producing CO_2_ (Table 1). The acid solutions used to be tested included 10% and 12% *w*/*w* HCl. Eight hundred grams of limestone was sufficient to produce the desired CO_2_ rate throughout the night. Three replicates were performed for each HCl acid concentration (each replicate per day (from 9 a.m. to 9 p.m.), six days in total). For each replicate, CO_2_ output was initially measured at one minute, shortly after dripping acid solution, and continued measuring occurred at the end of each hour for 12 h. CO_2_ output was measured as the volume of water displaced in a submerged measuring cylinder, as mentioned previously.

### 2.3. Field Evaluation

The CO_2_ generating system was evaluated in the field from June to November 2020 in three rural villages which are about 1–3 km apart, Ban Pa Chi (18.60296 N 98.83902 E), Ban Hua Rin (18.5959 N 98.84416 E) and Ban Pa Oi (18.584779 N 98.850397 E) of Sanpatong District, Chiang Mai Province (approximately 35 km from Chiang Mai city), Thailand. The study areas were selected based on accessibility in all seasons, availability of rice fields, animal sheds as blood sources and potential breeding habitats of *Anopheles*, *Aedes*, *Culex* and *Mansonia* mosquitoes.

#### 2.3.1. Experimental Design

**Figure 3:**
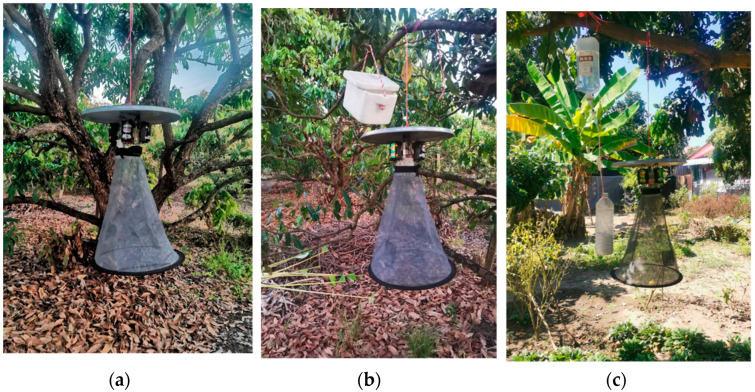
Three sets of traps in this study: (**a**) light trap (Set I); (**b**) light trap with dry ice (Set II); (**c**) light trap with CO_2_ from limestone and acid reaction (Set III).

**Figure 4:**
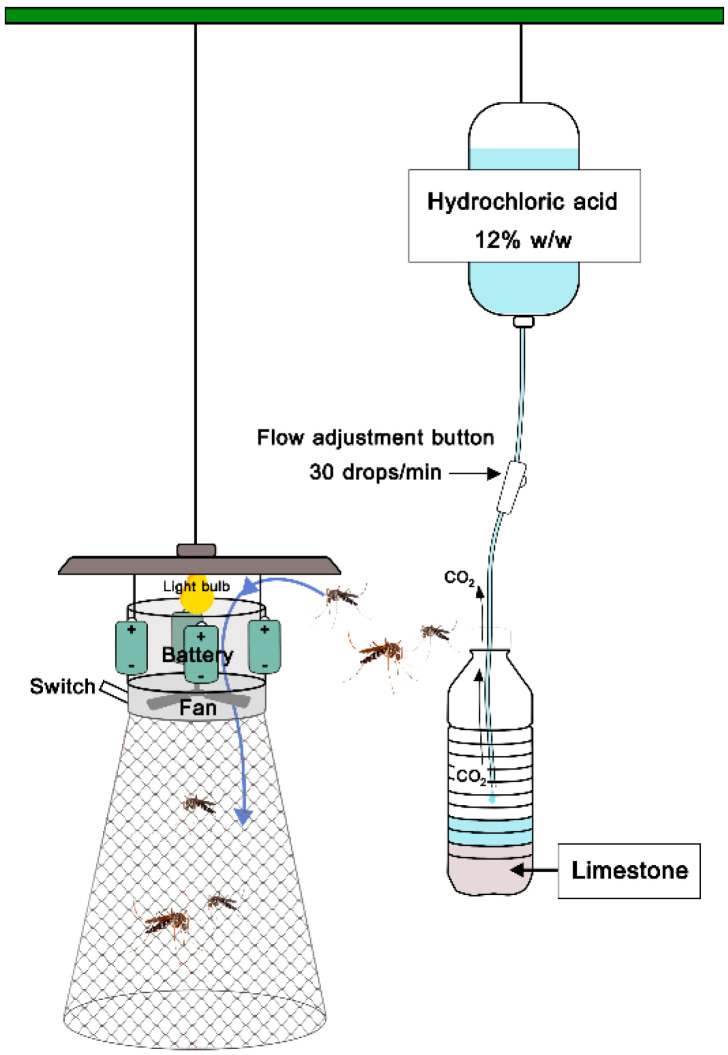
CO_2_ generating system from limestone and acid solution.

Mosquitoes were collected by three sets of traps: Set I: light trap only, Set II: light trap with dry ice (1 kg) and Set III: light trap with limestone (800 g) + 12% *w*/*w* HCl (1 L) (Figure 3). Light traps in this study were locally made and mimicked CDC light traps. They were operated by 4 × 1.5 volt batteries. Extraneous variation between treatments is time (day) and trap location, with the number of treatments being equal. In each village, mosquitoes were collected at three fixed sites (about 30–50 m apart), each with one of the three sets of traps. To reduce bias, a Latin square design was applied for mosquito collection. The rotation scheme for the three treatments (trapping) was allocated in a 3 × 3 arrangement for each trial replicate (Table S1). The trap sets were rotated daily for three rounds (9 nights/village or 27 nights in total). The traps were hung 1.5 m above the ground. A dry ice box and limestone + acid-generated CO_2_ bottles were placed slightly above the traps (Figure 3 and Figure 4). Collections started at dusk and ended at dawn. Ambient air temperature and relative humidity were recorded each collection day using a digital hygrothermometer (Union TH-02C, Sang Chai Meter, Bangkok, Thailand). Accumulated rainfall (mm) data were obtained from the Thai Meteorological Department.

#### 2.3.2. Mosquito Identification and Dissection

Captured mosquitoes in bags were transferred to the laboratory for counting and morphological identification using a stereomicroscope, following the standard keys of Rattanarithikul et al. [14] for *Culex*, Rattanarithikul et al. [15] for *Anopheles*, Rattanarithikul et al. [16] for *Mansonia* and *Coquillettidia*, and Rattanarithikul et al. [17] for *Aedes* and *Armigeres* (tribe Aedini).

Due to the presence of acid vapor in the CO_2_ generating system, which might affect the behavior of female mosquitoes with different age structures, the parity rate was determined. After identification, females of the three most abundant species collected from each trap set were randomly selected, and their ovaries were dissected to determine parity, as described by Detinova [18]. With the aid of entomological needles, the ovaries were separated from the rest of the surrounding tissues, placed in a drop of distilled water, allowed to be air-dried and examined under a compound microscope. Parous and nulliparous mosquitoes were classified by observing the coiling of the tracheoles [19].

### 2.4. Statistical Analysis

The numbers and species of mosquitoes collected from the three sets of traps in the three villages were analyzed by negative binomial regression. Differences in the number of parous females from each trap and location were analyzed by Pearson’s chi-squared test. Others were indicated in experimental methods or results. Analyses were made using SPSS version 22.0 (IBM Corp., Armonk, NY, USA).

## 3. Results

### 3.1. CO_2_ Production from Limestone

The amounts of gas produced from 1 g of limestone compared with 1 g of calcium carbonate reacted with excess H_2_SO_4_ (15% *w*/*w*) and HCl (10% and 12% *w*/*w*) solutions are shown in Table 1. One gram of calcium carbonate reacted with excess 15% H_2_SO_4_ produced an average of 236 mL CO_2_, which was about 16% and 9% lower than that produced by 10% and 12% HCl solutions, respectively. Similarly, the yield of CO_2_ gas obtained from 1 g limestone reacted with 15% H_2_SO_4_ solution (average 184 mL) was about 29% and 28% lower than 10% and 12% HCl solutions, respectively. Compared with calcium carbonate, limestone produced less CO_2_ gas, about 22% when reacted with 15% H_2_SO_4_ and 8% and 2% when reacted with 10% and 12% HCl solutions, respectively.

### 3.2. CO_2_ Generating System for Overnight Collection

**Figure 5:**
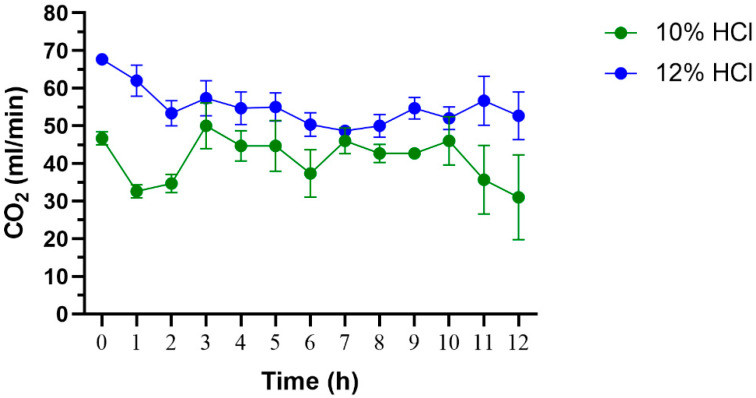
The amount of CO_2_ produced from limestone and dripping acid solutions (30 drops/min) measured at one-hour intervals. Mean and standard error are indicated.

Figure 5 shows the output of CO_2_ produced at one-hour intervals. CO_2_ gas was detected shortly after dripping acid solution onto the limestone and was continuously produced for up to 12 h. The average output of CO_2_ gas generated by 10% HCl (41.13 ± 2.19 mL/min) was significantly lower than 12% HCl (55.00 ± 1.64 mL/min) (*t*-test, *p*-value = 0.0071).

### 3.3. Field Evaluation

A total of 1620 mosquitoes collected from the three villages by the three different trap sets for 27 nights are summarized in Table 2. Six mosquito genera, *Aedes*, *Anopheles*, *Armigeres*, *Coquilletidia*, *Culex* and *Mansonia*, were captured, consisting of 1583 (97.7%) females and 37 (2.3%) males. *Culex* mosquitoes were the most abundant in all study locations. The predominant species collected were *Culex vishnui* (*n* = 760, 46.91%), *Cx. bitaeniorhynchus* (*n* = 504, 31.11%) and *Cx. tritaeniorhynchus* (*n* = 157, 9.69%). The numbers of mosquitoes collected by the three trap sets were statistically different (*p*-value < 0.001). Set II (light trap *+* dry ice) collected the highest number of mosquitoes (total 1341) and species (14 spp.), followed by Set III (light trap *+* limestone *+* acid) (total 267 and 9 spp.). Mosquitoes collected by Set I (light trap only) yielded the lowest number (total 12) and species (6 spp.). Although Set III yielded fewer mosquitoes than Set II, it collected all species of *Aedes*, *Culex*, *Coquillettidia* and *Mansonia* as found in Set II. *Armigeres* and *Anopheles* mosquitoes, which were low in prevalence during the time of collection, were not collected by Set III. When comparing the number of mosquitoes obtained between three study villages, overall, Ban Pa Oi showed significantly fewer than the other two villages (Table S2). This fewer number might be affected by the lower temperature and more rainfall during October–November, which this period is the late rainy season and early winter in Thailand (Table S3). The temperature, relative humidity and accumulated rainfall during the study period were shown in Table S3.

The parity rates were determined in three *Culex* species, *Cx. quinquefasciatus*, *Cx. vishnui* and *Cx. bitaeniorhynchus*, which were randomly selected from trap Set II and trap Set III (Table 3). Pearson’s chi-squared test showed no statistical difference (*p*-value > 0.5), indicating that there was no bias in the collection of mosquitoes with different physiological ages. Trap Set I was not included in this Pearson’s chi-squared test because the number of mosquitoes obtained were too low (<5 females per species).

## 4. Discussion

In this study, we developed a limestone and acid combination system that reliably produces CO_2_ throughout the night, and can be incorporated with CDC light traps for mosquito sampling. Instead of using weak acids that require a large volume of aqueous acid solution to produce sufficient CO_2_ [9,10], we used HCl which can minimize the water volume needed to prepare the acid solution. The cost of the carbonate source in this study was also reduced by using limestone, which is inexpensive, widely available and contains a high quantity of calcium carbonate. The flow of CO_2_ is simply controlled by using an intravenous injection set, which is used routinely in hospitals for continuous administration of saline solution or medicines. Hence, no sophisticated instruments are needed in our technique. In Thailand, the prices of 37% HCl acid (2.5 L) and limestone (50 kg) are about 20 USD and 2 USD, respectively. Therefore, the cost of one set of this CO_2_ generating system (12% HCl (1 L) and limestone (800 g)) is about 2–3 USD per night of collection. Used intravenous injection sets (needle removed) and plastic bottles (1.5–2 L) may be obtained from a hospital without cost. It is long-lasting and can be reused many times without problems.

Laboratory and field experiments revealed that the technique outlined in the present study is reliable in producing the desired CO_2_ output (50–70 mL/min) for over 12 h. Compared with the unbaited trap (without CO_2_) (Set I), trap Set III (limestone + acid) collected 22-fold more mosquitoes and greater species composition. This may be explained by the fact that trap Set I was placed far from human houses or animal shelters, and there were many light sources in the study villages, which may compete with the light from the trap. As expected, the trap with dry ice as bait (Set II) collected the highest number of mosquitoes (1341), about five times greater than that collected from Set III. The number of mosquitoes collected appeared to increase with the concentration of CO_2_. The amount of CO_2_ gas generated from 1.5 kg of dry ice per 351 mL/min [5], while CO_2_ generated from the reaction of limestone and HCl (12% *w*/*w*) in this study was 55 mL/min on average (or about four times less than that generated in trap Set II). Nonetheless, all of the important species in the study villages, which are vectors of viral diseases and/or filariasis [20,21] collected by Set II were also collected by Set III, i.e., *Ae. aegypti*, *Cx. bitaeniorhynchus*, *Cx. gelidus*, *Cx. quinquefasciatus*, *Cx. tritaeniorhynchus*, *Cx. vishnui*, *Cq. crassipes* and *Mansonia uniformis*. However, *Anopheles* spp. and *Armigeres subalbatus*, which were zoophilic and very rare (only 1%–2% of the total number), were not collected by Set III. This might be explained because the flow rate of CO_2_ released from limestone and acid was lower than dry ice. In accordance with McPhatter and Gerry [22], the level of CO_2_ release rate was significantly correlated with mosquito capture rate. Further study is needed for sampling *Anopheles* malaria vectors in endemic areas.

As an increased amount of CO_2_ enhances the efficacy of trap collection, the quantity of CO_2_ generated from combining limestone and HCl solution as outlined in the present study could be increased as follows: (1) use more than one CO_2_ generating system per trap; (2) increase the amount of limestone and the volume of acid solution delivered per min; (3) increase the concentration of acid solutions. However, the latter is not recommended because of the dangers of handling higher acid concentrations. Calcium chloride (CaCl_2_), the product of the reaction, is well known as a de-icing and dust control compound. This substance is highly soluble in water and generally recognized as safe (GRAS) by the U.S. Food and Drug Administration. One disadvantage of our method is the use of a strong acid. To reduce the hazard, carrying diluted acid solutions to the field is recommended. In addition, the concentration of HCl aqueous solution may be reduced. Accordingly, there is a need to use larger bottles for both acid solution and limestone. In addition, we experienced that limestone powder available in markets is variable in quality affecting the amount of generating CO_2_, and product that had been stored for a long time showed a significant loss of CO_2_ production. Therefore, it is necessary to perform a quantitative analysis to determine generated CO_2_ in the laboratory before using it in the field. Alternative inexpensive carbonate materials, e.g., marble, chalk, mollusk shells and eggshells, composed of high calcium carbonate, could be used depending on local availability. Of course, they need to be analyzed for CO_2_ production in the laboratory before being used in the field.

## 5. Conclusions

The CO_2_ generating system by combining limestone and HCl solution in the present study is reliable, simple and inexpensive. When incorporated with a CDC light trap, it enhances the efficacy of collecting mosquitoes compared with the unbaited trap. Although the number of caught mosquitoes was lower than that used with dry ice, there was little effect on the species composition of mosquitoes in the study areas. Apart from nighttime biting mosquitoes, this technique could be tested for collecting daytime biting mosquito species (e.g., *Ae. aegypti*) and other blood-sucking insects and arthropods.

## Figures and Tables

**Table 1:** Amount of CO_2_ gas produced by the reaction between 1 g of limestone and excess acid solution, compared with 1 g of calcium carbonate.

Substrate	Excess H_2_SO_4_ Solution	Excess HCl Solution	CO_2_ Gas (mL)	Average ± S.D. (mL)
CaCO_3_ 1 g	15%		238	236 ± 2.8
			234	
		10%	282	281 ± 1.4
			280	
		12%	265	260 ± 7.1
			255	
Limestone 1 g	15%		190	184 ± 3.0
			183	
			180	
		10%	260	259 ± 1.4
			258	
		12%	255	256 ± 1.4
			257	

**Table 2:** Numbers and species of mosquitoes collected from three locations by three different trap sets in Sanpatong District, Chiang Mai Province, Thailand.

Mosquitoes Species	Set I (Light Trap)		Set II (Light Trap + Dry Ice)		Set III (Light Trap + Limestone + HCl)		Total (%)
	F	M	F	M	F	M	
*Aedes aegypti*	0	0	1	0	2	2	5 (0.31)
*Armigeres subalbatus*	1	0	14	0	0	0	15 (0.93)
*Anopheles barbirostris* s.l.	0	0	4	0	0	0	4 (0.25)
*Anopheles hyrcanus* group	0	0	10	0	0	0	10 (0.62)
*Anopheles vagus*	0	0	2	0	0	0	2 (0.12)
*Anopheles tessellatus*	0	0	2	0	0	0	2 (0.12)
*Coquillettidia crassipes*	0	0	22	0	2	0	24 (1.48)
*Culex bitaeniorhynchus*	4	0	412	15	71	2	504 (31.11)
*Culex gelidus*	0	1	8	1	4	2	16 (0.99)
*Culex nigropunctatus*	0	0	10	0	3	0	13 (0.80)
*Culex quinquefasciatus*	1	0	24	0	30	2	57 (3.52)
*Culex tritaeniorhynchus*	1	0	114	1	41	0	157 (9.69)
*Culex vishnui*	4	0	649	5	101	1	760 (46.91)
*Mansonia uniformis*	0	0	42	5	4	0	51 (3.15)
Total	11	1	1314	27	258	9	1620

**Table 3:** Parity rate of *Culex quinquefasciatus*, *Cx. vishnui* and *Cx. bitaeniorhynchus* collected from trap Set II and trap Set III.

Mosquitoes Species	Ovary Status	Set II (Light Trap + Dry Ice)	Set III (Light Trap + Limestone + HCl)
*Culex quinquefasciatus*	Nulliparous	7 (29.2%)	11 (36.7%)
	Parous	17 (70.8%)	19 (63.3%)
	Total	24	30
	Pearson’s chi-squared 0.338 (*p*-value = 0.772)		
*Culex vishnui*	Nulliparous	236 (36.4%)	43 (42.2%)
	Parous	413 (63.3%)	59 (57.8%)
	Total	649	102
	Pearson’s chi-squared 1.267 (*p*-value = 0.272)		
*Culex bitaeniorhynchus*	Nulliparous	163 (49.6%)	29 (42.0%)
	Parous	249 (60.4%)	40 (58.0%)
	Total	412	69
	Pearson’s chi-squared 0.150 (*p*-value = 0.791)		

## Data Availability

The data presented in this study are available in the article or Supplementary Materials.

## Supplementary Materials

The following supporting information can be downloaded at: https://www.mdpi.com/article/10.3390/insects13070637/s1, Table S1: Latin square rotation scheme adopted for evaluating three mosquito trap sets in each village; Table S2: Comparison of the number of mosquitoes obtained from each study location; Table S3: Meteorological information in the study locations during the study period.
