# Supplementary material for: A Simple CO2 Generating System Incorporated with CDC Light Trap for Sampling Mosquito Vectors
Source: Insects. 2022 Jul 15;13(7):637. doi: 10.3390/insects13070637 (PMC9322468; doi:10.3390/insects13070637)
Supplement: Supplementary file 1 [file insects-13-00637-s001.zip › insects-1785962-supplementary.pdf]

Supplementary Materials

Table S1. Latin square rotation scheme adopted for evaluating three mosquito trap sets in each village.

Day	Site 1	Site 2	Site 3
1	Light trap	Light trap with dry ice	Light trap with CO_2 from the chemical reaction
2	Light trap with CO_2 from the chemical reaction	Light trap	Light trap with dry ice
3	Light trap with dry ice	Light trap with CO_2 from the chemical reaction	Light trap

Table S2. Comparison of the number of mosquitoes obtained from each study location.

Trap	Number of mosquito/trap night (Mean ± S.E.)		
	**Ban Pa Chi**	**Ban Hua Rin**	**Ban Pa Oi**
Light trap [1]	1.10 ± 0.59 [a]	0.11 ± 0.11 [a]	0.11 ± 0.11 [a]
Light trap + dry ice [2]	61.0 ± 10.00 [a]	74.00 ± 21.00 [a]	13.00 ± 2.90 [b]
Light trap + limestone + HCl [2]	18.00 ± 7.30 [a]	10.00 ± 2.90 [a,b]	1.90 ± 0.54 [b]
All trap sets [2]	27.00 ± 6.40 [a]	28.00 ± 9.40 [a]	5.10 ± 1.50 [b]

The same superscript letters in each row indicate no significant difference (p-value > 0.05).
[1] Kruskal-Wallis test, followed by Dunn's multiple comparison test
[2] ANOVA, followed by Bonferroni's multiple comparison test

Table S3. Meteorological information in the study locations during the study period.

Study duration	Location	Average temperature (°C) (Min – Max)	Average humidity (%) (Min – Max)	Accumulative rainfall (mm.) [1]
June - July 2020	Ban Pa Chi	27.4 (24 – 33)	77.0 (48 – 93)	1.8
August - September 2020	Ban Hua Rin	26.6 (23 – 31)	80.2 (65 -95)	14.8
October - November 2020	Ban Pa Oi	25.2 (17 – 31)	75.3 (51 – 91)	168.2

[1] Source: rain fall (http://hydromet.tmd.go.th/Reports/report-rf-province-month.as)
